# Advancements in robotic arm-based 3D bioprinting for biomedical applications

**DOI:** 10.1093/lifemedi/lnad046

**Published:** 2023-11-21

**Authors:** Kai Li, WenHui Huang, HaiTao Guo, YanYan Liu, Shuxian Chen, Heng Liu, Qi Gu

**Affiliations:** State Key Laboratory of Membrane Biology, Institute of Zoology, Chinese Academy of Sciences, Beijing 100101, China; Savaid Medical School, University of Chinese Academy of Sciences, Beijing 101499, China; State Key Laboratory of Membrane Biology, Institute of Zoology, Chinese Academy of Sciences, Beijing 100101, China; Savaid Medical School, University of Chinese Academy of Sciences, Beijing 101499, China; State Key Laboratory of Membrane Biology, Institute of Zoology, Chinese Academy of Sciences, Beijing 100101, China; Savaid Medical School, University of Chinese Academy of Sciences, Beijing 101499, China; School of Materials Design and Engineering, Beijing Institute of Fashion Technology, Beijing 100029, China; State Key Laboratory of Membrane Biology, Institute of Zoology, Chinese Academy of Sciences, Beijing 100101, China; Savaid Medical School, University of Chinese Academy of Sciences, Beijing 101499, China; State Key Laboratory of Membrane Biology, Institute of Zoology, Chinese Academy of Sciences, Beijing 100101, China; Department of Orthopaedics, Beijing Jishuitan Hospital Affiliated to Capital Medical University, Beijing 100035, China; State Key Laboratory of Membrane Biology, Institute of Zoology, Chinese Academy of Sciences, Beijing 100101, China; Savaid Medical School, University of Chinese Academy of Sciences, Beijing 101499, China; Bioinspired Engineering Group, Beijing Institute for Stem Cell and Regenerative Medicine, Beijing 100101, China

**Keywords:** 3D bioprinting, robot arm-based bioprinting, tissue engineering, *in situ* bioprinting, hydrogel

## Abstract

3D bioprinting emerges as a critical tool in biofabricating functional 3D tissue or organ equivalents for regenerative medicine. Bioprinting techniques have been making strides in integrating automation, customization, and digitalization in coping with diverse tissue engineering scenarios. The convergence of robotic arm-based 3D bioprinting techniques, especially *in situ* 3D bioprinting, is a versatile toolbox in the industrial field, promising for biomedical application and clinical research. In this review, we first introduce conceptualized modalities of robotic arm-based bioprinting from a mechanical perspective, which involves configurative categories of current robot arms regarding conventional bioprinting strategies. Recent advances in robotic arm-based bioprinting in tissue engineering have been summarized in distinct tissues and organs. Ultimately, we systematically discuss relative advantages, disadvantages, challenges, and future perspectives from bench to bedside for biomedical application.

## Introduction

To meet the escalating urgency for organ transplantation, tissue engineering and regenerative medicine have emerged as a prevailing field for organ fabrication. In this landscape, 3D bioprinting is utilized to fabricate 3D living constructs, such as blood vessels, heart, cartilage, and liver, in a layer-by-layer fashion via precisely depositing biomaterials, living cells, and growth factors [1]. In this case, 3D tissues and potentially living organs can be fabricated for various biomedical applications, including drug screening [2] and disease modeling [3]. With the orchestration of novel bioprinting modalities such as freeform reversible embedding of suspended hydrogels (FRESH), scaffold-free bioprinting, and stereolithographic apparatus for tissue engineering (SLATE), constructing large-scale organs with controlled vascularization and favorable resolutions is achievable for better mimicking native tissues *in vivo* [4, 5].

Robotic arms are required to precisely position certain materials with complicated shapes and curved surfaces, which is labor-saving for reproductivity and scalability. Therefore, versatile robotic arms have been extended in medical applications such as DOBOT in the healthcare industry for medical device packaging, lab testing, and blood sampling with affordability and accessibility. Nowadays, medical robots are relatively prevalent in surgery, which aims to deploy surgical facilities for medical procedures via the synergy of robotic arms and software. For instance, for neurosurgery, Modus V, a robot arm, is employed to track surgical instruments while projecting operating images with high resolution [6]. Inspired by the medical-specified robotic arm, 3D bioprinting technology has nourished from the advantageous flexibility. Innovative 3D bioprinting techniques, specifically *in situ* 3D bioprinting, have been applauded for directly depositing living cells, biomaterials, and biochemicals to lesion sites since 2007 [7]. Incorporated with robotic arms, *in situ* 3D bioprinting comprises intricate shapes and curved surfaces compared with conventional 3D bioprinting patterns where bioink is dispensed on plain substrates [8]. Furthermore, robotic arms with cartesian, cylindrical, polar/spherical, articulated, and delta configurations have been employed in organ fabrication. Progressive developments in organ regeneration combined with robotic arms and 3D bioprinting have been verified in various studies, such as skin, muscle, cartilage, and bone [9].

**Figure 1.**
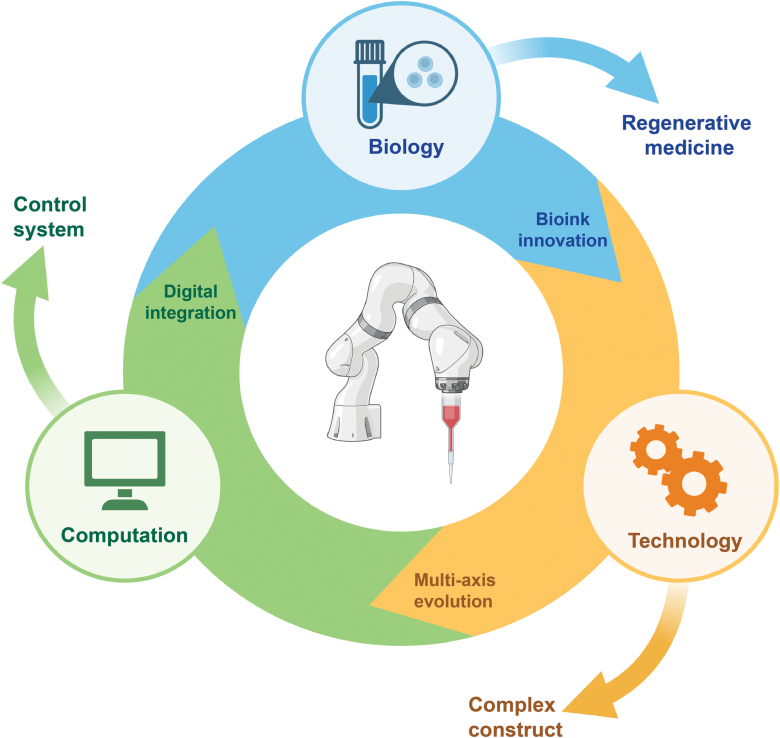
Comprehensive overview of robotic arm-based 3D bioprinting in biomedical applications, including configurative modalities of robotic arms, tissue engineering cases, existing challenges, and future perspectives.

In this review, we comparatively recapitulate current robotic arm-based bioprinting modalities from the mechanical perspective. Typical approaches and cutting-edge techniques for *in situ* 3D bioprinting are discussed. What’s more, robotic-arm-based 3D bioprinting in tissue regeneration is relatively illustrated. Moreover, commercial bioprinters based on dimensional configurations are addressed. We also concisely explore the current challenges and possible future orientations regarding robotics, organ regeneration, and personalized medicine (Fig. 1).

## Robotic arm-based 3D bioprinting

Robotic arm-based 3D bioprinting technologies emerge as a pivotal tool in tissue engineering and regenerative medicine with high flexibility compared with conventional 3D bioprinting modalities. Relative differences have been summarized in Table 1. Robotic arm-based strategies exhibit great potential in *in situ* bioprinting, especially for biomedical applications. Diverse robotic arms are exploited to meet specific tissue engineering and regenerative medicine demands. Despite the dominant merits of robotic arm-based 3D bioprinting in customized design and printing efficiency regardless of cell pre-loading and tissue culture, it is vague in elucidating applicable efficacy for tissue regeneration. Nowadays, various robotic arms are built with essential core capabilities, especially well-matched for specific roles or application contexts. Robotic arms typically feature up to six joints interconnecting seven segments, powered by distinctive stepper motors and controlled by computers.

**Table 1.** General comparison of 3D bioprinting techniques.

Bioprinting types	Printing pattern	Cost	Cell viability	Printing speed	Bioink viscosity	Resolution	Ref.
Inkjet	Drop by drop	Low	>85%	Fast	3.5–12 mPa/s	50 μm	[10]
Laser	Dot by dot	High	>95%	Medium	1–300 mPa/s	10 μm	[11]
Extrusion	Line by line	Medium	50–90%	Slow	Up to 6 × 10^7^ mPa/s	100 μm	[12]
Stereolithography	Projection-based	Low	>85%	Fast	No limitation	25 μm	[12]
Robotic arm-based	Six-degree-of-freedom	High	>75%	Fast	Up to 6 × 10^7^ mPa/s	100 μm	[9]

**Figure 2.**
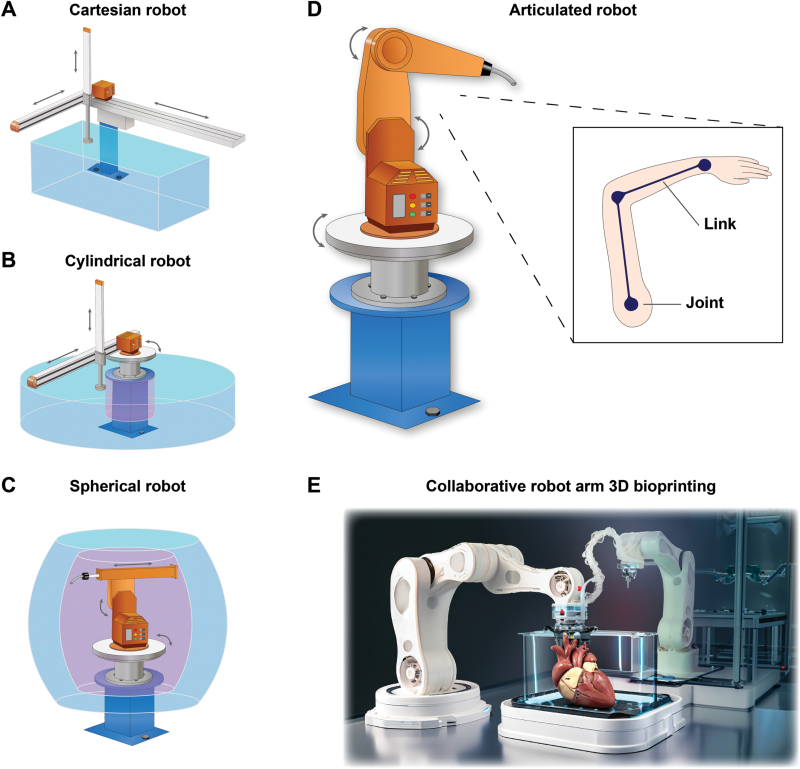
**Configuration of serial link robot arms for 3D bioprinting.**(A) Cartesian robots. (B) Cylindrical robots and (C) spherical robots both have a dead zone in their central area. (D) Articulated robots. (E) Diagram of collaborative work pattern of robotic arm-based 3D bioprinting.

*De facto*, robotic arms and humans’ share the same underlying structure of links and joints. Parts that can freely bend and move about, such as the elbow and shoulder, are the joints, and the bones connecting those joints are equivalent to a robot’s links (Fig. 2D). Both humans and robots work on the same idea of moving joints and transferring power through linkages. According to how their links are organized, robots can be divided into two categories: parallel link and serial link. Due to the alignment of their joint affinity, delta robots are categorized as a parallel link. On the contrary, cartesian, cylindrical, polar/spherical, and articulated robot arms are classified as serial link since their joints are aligned in series.

Consequently, this enables extraordinarily precise emplacement of the “hand”, typically a specialized tool or end effector, to perform a specific action or a recurrent sequence of articulations. Given a mechanical perspective, dimensional robotic arm-based systems have contributed to certain bioprinting cases. Specified configurations of typically utilized serial robotic arms are summarized in Fig. 2, and accordingly, comparisons of distinct configurations are discussed in Table 2.

**Table 2.** Comparisons of robotic arm-based configurations for 3D bioprinting.

Configuration	Advantages	Disadvantages
Delta	1. High-speed and acceleration 2. High portability	1. Suitable for moderately heavy loads
Cartesian	1. Simple design and operation 2. High accuracy 3. Affordable	1. Large volume of workspace 2. Lack of flexibility 3. Limited portability
Cylindrical and spherical	1. High-load carrying capability	1. Take up more space
Articulated	1. High speed 2. Greater flexibility	1. High cost 2. Complicated configuration

### Configurations

Robots are designed based on the “work envelope”, or the volume that the end effector of the robot arm can access. The envelope of a robot is determined by three physical properties. The first is the robot’s joint range, which comprises linear and angular ranges. The robot’s physical size, which is the second characteristic, will create a dead zone and reduce the proportion of the operating envelope within the robot’s work envelope. The final factor is the type of joint: rotary or linear. Various robot configurations have a distinctive work envelope. The particular application requirement affects what kind of robot setup is needed.

#### Delta robotic arm

Delta robotic arms, also referred to as “parallel robotic arms”, since they enable a single base installed over a workspace to support multiple arms (usually three) [13]. These robot arms are employed for automation featuring high-speed alternatives. Additionally, they are equipped with a unique dome-shaped construction to enable fine-tuned, accurate movements at high velocities since each of the three arms independently controls a different end effector joint [14]. Thus, delta robotic arms are a promising option in biomedical industries. As for 3D bioprinting, it has been reported that a delta robotic arm-based bioprinter could be applied to extrude cell-laden hydrogels *in situ* on mice for studies of wound-healing diseases, which is also a promising avenue for the development of bodily printed smart wearable devices [15]. More interestingly, since the uniqueness of the delta robotic arms, researchers have developed a micro bioprinting platform for *in vivo* bioprinting at a gastric wound site, where the delta robot flexibly folds and unfolds throughout the bioprinting process [16].

#### Cartesian robotic arm

Cartesian robotic arms, also known as gantry robotic arms, are composed of three articulating joints designed to move linearly in three dimensions utilizing *X*, *Y*, and *Z* coordinates (Fig. 2A). Further rotary capability is frequently provided by the wrist joint. Cartesian robotic arms are based on motors and actuators to 3D manipulate printing tools, whose movements are restricted to a linear manner horizontally and vertically. Current 3D bioprinters commonly adapt cartesian coordinate robotic arms in extrusion-based and inkjet bioprinting. Despite its broad application in conventional 3D bioprinting modalities, it is incapable of printing on anisotropic or curved surfaces. Besides, the operative procedure lacks flexibility; the workspace tends to be restricted. To address this dilemma, researchers have developed a hybrid method integrating cartesian and soft robotic arm bioinspired by “tendon cable” for *in situ* bioprinting to achieve motion in six independent degrees of freedom (DOF) [17].

#### Cylindrical robotic arm and spherical robotic arm

Contrary to the above-described cartesian versions, cylindrical robot arms are those whose axes create a cylindrical coordinate framework. Replace one linear joint from the cartesian robotic arm with a rotary joint, and the robot arm can rotate 360 degrees on its central axis. The cylindrical robot arm is connected to the base by a twisting joint, in which case, polar motion is accomplished via coupled rotational joints, rotary joints, and a linear joint (Fig. 2B). In essence, the preprogrammed movements occur in a cylinder-shaped manner (up, down, and around). The rotary and prismatic joints on cylindrical arms allow it to move in both rotational and linear motion, making it primarily employed in automation systems as intermediaries between two or three mechanical devices to move an object from one location to another. Cylindrical robotic arms are also frequently utilized for assembly assignments and machine tool handling.

However, the robot must turn at different complicated angles for tasks like welding car chassis together and painting on a curved surface. As a result, the cylindrical robot cannot complete this task; instead, a more versatile robot arm is required. By substituting a linear joint from the cylindrical robot arm for one extra rotary joint, the spherical or polar robotic arm can accomplish this. In the central area of the partially spherical work envelope, the spherical robot arm also exhibits a dead zone similar to the cylindrical robot arm, which is an efficiency weakness (Fig. 2C).

As for the uniqueness of cylindrical and spherical robotic arms, it could be foreseen in the future that they might be an effective tool for *in situ* 3D bioprinting in cylindrical and partially spherical shaped tissues or organs, such as the colon, intestines, and uteri, in circumstances without the need to add more axes. In this case, the aforementioned robotic arms could be a more efficient and simpler solution than articulated robot arms to cope with this issue.

#### Articulated robotic arm

Adding another rotational joint in place of the last linear joint of the spherical robotic arm will make it a revolute coordinate robot, which diminishes the dead zone and provides more flexibility (Fig. 2D). A human-like robot arm with motorized motion and configuration is referred to as an articulated robotic arm. It corresponds to the most prevalent categories of robotic arms employed in manufacturing automation, composed of a single robotic arm twistingly coupled to a base. The most widespread type of articulated robot is a multi-axis robot, which typically has four to six axes, enabling a more excellent range of mobility. In this case, bioink could be deposited in a non-linear plane with a complex topological profile from any DOF [18]. Moreover, this highly humanoid technique benefits from being portable, which minimizes its environmental impact [19]. In the *in situ* operative scenario, articulated robotic arms could be indispensable in the scale of delicacy, especially in neurosurgery [20].

Another type of articulated robotic arm is Selective Compliance Articulated Robot Arms, known as SCARA robotic arms, which are broadly employed in assembly and pick-and-place scenarios. The phrase “selective compliance” refers to the arm’s minor compliance in the *X*–*Y* direction but rigidity in the Z direction due to the parallel-axis joint configuration of the SCARA. This is conducive for many kinds of assembly tasks, such as inserting a round pin without binding in a round hole. SCARA 3D bioprinting is preferable when a limited degree of flexibility is required, which is well-suited for the printing task in particular directions.

Additionally, articulated robots’ controlling interface and programming procedure are more intricate than cartesian robots. For example, by adding an extra robot arm to accomplish more complicated 3D bioprinting tasks in a cooperative manner (Fig. 2E). On the other hand, the addition of axes can be leveraged to enhance manipulability while achieving more nuanced motions, although it could render inverse kinematics more challenging [21]. As for robotic arm-based bioprinting, it is demonstrated that intraoperative 3D bioprinting could be feasible to treat deep third-degree severe burn injuries via six-axis robotic arms [22]. Li *et al*. also employed a robotic manipulator 3D printer in a swine model, validating the practicality of repairing large-segmented bone defects [23].

### Operational mechanism of robotic arm-based bioprinting

**Figure 3.**
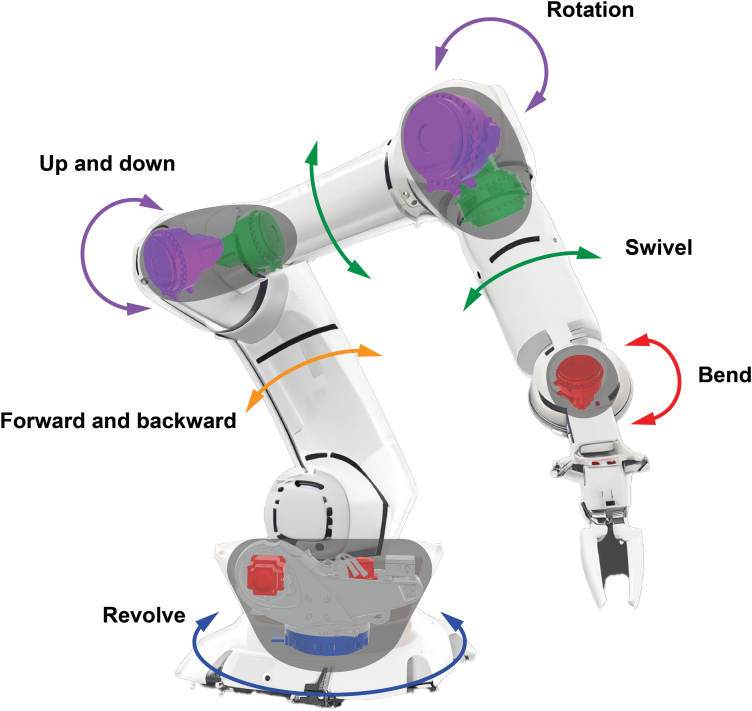
Illustration of the articulated robotic arm-based 3D bioprinter internal structure and indications of joints and linkages.

The robot is capable of operating in a confined location since interference with peripheral equipment can be avoided, and wires and harnesses can be compacted inside the hollow arm, which suits well for a clean, hygienic environment. Automation reduces the risk of exposure to harmful chemicals and contamination. It distinguishes itself by operating quickly and permitting nimble motions, whose nature could be coupled well as a 3D bioprinter (Fig. 3).

Typically, a robot arm consists of distinct parts. Among those parts are four significant ones: the actuator, reduction gear, transmission system, and encoder.

The actuator is a part that serves as the robot’s joint. This component transforms energy into mechanical motions, enabling a robot’s arm to move up and down or swivel. Servo motors, which have the capability to adjust position and speed to carry out movements with great precision, are typically employed as actuators in robot arms. Nevertheless, the amount of power that a motor can produce on its own is constrained. Motors are applied in conjunction with a reduction gear to produce additional power. Subsequently, adjusted power generated by the actuators and reduction gears is transmitted by the transmission system, such as belts and gears. The transmission system can also change the direction and magnitude of power. With the flexibility of the conduction mechanism, robots have a compact structure since a motor could be placed away from the joints, such as installed on the elbow part of the robot arm. Bicycles, for instance, use a transmission to take the rotational movement from the pedals and transmit it to the back wheel through a variety of different-sized gears. The rear wheel’s gears are often changed via the gearbox (transmission system), which connects the crank to the back wheel. Choosing a large gear reduces the amount of wheel spins, which makes pedaling easier but reduces speed. It becomes considerably simpler to ride up steep hills, and output power can be elevated. This is the same operational mechanism as reduction gears in robot arms.

Additionally, the robot arm should be equipped with an encoder to indicate the location and rotating angle of a motor. Data are obtained by general optical encoders from a disk that is fastened to the motor’s rotational shaft. The disk features perforations that allow light to pass through at regular intervals. On both sides of the disk, there are light-emitting diodes and light-receiving components (photodiodes) which can distinguish between different light intensities (light and dark). Reading the signals allows one to identify the rotation angle and speed of the motor for the reason that the light is either blocked or passes through the slits as the motor rotates. An encoder can transfer precise information on the amount and direction of the robot’s movement. Servo motors can control placement and speed precisely as a result.

## Advantages of robotic arm-based 3D bioprinting

Conventional extrusion-based 3D bioprinters use digital design files that are uploaded to the device and converted into actual physical dimensions. The extrusion head is attached to a three-axis system, which is a cartesian robotic arm that enables movement across the *X*, *Y*, and *Z* axes while depositing bioink across a build platform layer by layer following a path specified by the design. The extrusion head travels up when the printer completes a layer, and the bioprinter starts working on the following layer. This process repeats until the part is finished. Several process parameters can be adjusted, comprising the nozzle, platform temperatures, print speed, extrusion pressure, layer height, and nozzle gauges.

Compared to a conventional extrusion-based 3D bioprinter, a cartesian robotic arm, the main difference of an articulated robotic arm multiple-axes extrusion-based 3D bioprinter is that it has more axes and rotary joints, which means higher flexibility in essence. This nature determined that both of them would encounter problems in common during the print process. However, with the increased flexibility and versatility, articulated robotic arm 3D bioprinter will have some advantages when dealing with certain problems.

### Adhesion issues in 3D bioprinting

**Figure 4.**
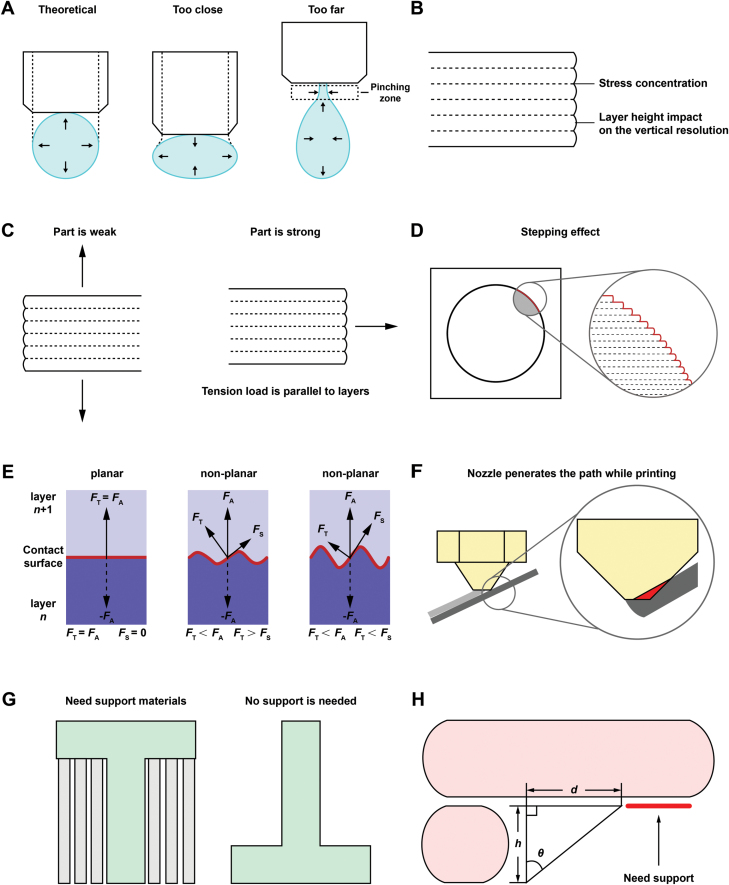
**Illustration of common technical limitations in 3D bioprinting.**(A) Extrusion profile of Fused Deposition Modeling (FDM). (B) Schematic of the layer-by-layer construction of 3D printed part. (C) The effect of part orientation on the strength of the printed parts. (D) Stepping effect of the curved surface with large curvatures. (E) Force distribution between layers of planar and non-planar surfaces. *F*_A_: attacking force; *F*_T_: tensile component of *F*_A_; *F*_S_: shearing component of *F*_A_. (F) The penetration issue during printing, where the nozzle pierces the printing path. (G) Illustration of support structures. (H) The triangle shows the maximum support angle *θ*, the layer height *h*, the maximum overhang distance *d*, and the area that needs support.

Secure adhesion between deposited layers of a part is critical in 3D bioprinting. When a bioprinter extrudes bioink through the nozzle, this material presses against the previously printed layer and adheres to each other; for some bioink, they will even melt together. In theory, the cross-section of the filament should be a perfect circle while printing. However, the print head should apply pressure to the filament of the bioink to keep the adhesion in addition to the properties of bioink materials, whose shape deforms to an oval. When the print head is too close, it will struggle to extrude the bioink and destroy the previous layer, causing the failure to achieve the actual layer and part height. On the contrary, when the print head is too far, it will generate adhesion problems. In addition, materials are stretched during the extrusion, causing deviations in filament diameter (Fig. 4A).

To solve this problem, 3D printers usually have a Z-probe detector to have a proper calibration of the first layer by changing the Z-offset. However, due to the nature of bioink materials, most of them are hydrogels, it is prone to be impractical for the bioprinter to overcome this obstacle with the same method. Not to mention, the properties of the hydrogel are easily changed by many factors during the printing process, such as environmental temperature and water evaporation. The deviations caused by the dynamic change of bioink during the printing process will superpose and bring about the incompleteness of the final structure. In tissues and organs, structure integrity is an indispensable factor in achieving certain biological functions such as the cornea [24], cardiac tissue [25], and vasculature system [26].

Conventional 3D bioprinters are “position control” systems that have a fixed path and constant print speed, giving rise to an uncontrolled contact force to the print surface, which is tricky to handle diverse changes of biomaterials during printing. Yet, with the adaptive force sensor applied in industrial robot arms nowadays, combined with the flexibility and versatility of multiple axes robotic arm-based 3D bioprinters, a system with an adaptive path and controlled contact force could be accomplished.

### Anisotropic control

Extrusion-based printing allows for the fabrication of bio-instructive scaffolds. Nonetheless, owing to the layer-by-layer nature of conventional 3D printing, layer height will have an impact on the vertical resolution of the part, affecting its smoothness. The effect of layer height is less pronounced on straight vertical walls and more pronounced on curves and angles. For instance, the printer software must slice the circular hole into numerous layers and stack them on top of one another in order to print a hole down a horizontal axis. This results in a non-smooth edge that resembles a staircase. This phenomenon, known as the “stepping effect,” is more evident on surfaces with higher curvature (Fig. 4D).

Most crucially, stress concentrations at the layer interface are brought on by notches between layers (Fig. 4B). In a cartesian 3D printing system, it will inherently alter the anisotropic properties of the printing parts, which imply that they are significantly more robust in the *X, Y* than the *Z* direction. It is vital to take the application and the load direction into account while designing functioning biomaterial structures. For instance, parts placed in tension in the *Z* direction instead of the *X, Y* direction are significantly more prone to delaminate and shatter (Fig. 4C). Besides the strength of 3D-printed parts, part orientation also affects the print time and quality in certain circumstances.

In recent research, conventional 3D bioprinting is restricted to shape mimicking, while the intrinsic alignment of native organs or tissues is often neglected in organ biofabrication. Thus, to accomplish a unique orientational profile of native tissues *in vivo*, it has been proven in previous research that alignment is vital for organ maturation and functionalization [27, 28]. In this case, robotic arm-based 3D bioprinting could be an empowered helper in tackling this challenge of orientation control at the macroscopic level.

### Customized printing contact angle

Non-planar printed parts are stronger than their planar counterparts. However, the different contact surface orientations between layers might result in a more consistent resistance to tensile stress. Delamination can occur in planar printed parts because they are often less tensile stress resistant along the build direction axis than along the other two dimensions. Resulting from the interlocking, non-planar layers can distribute tensile forces into a compound of tensile and shearing forces. The graph illustrates how, as displacement increases, the shearing component *F*_S_ rises (Fig. 4E).

Nevertheless, a conventional cartesian 3D printer’s nozzle would easily become entangled in infill structures and penetrate previously printed material when printing curved surfaces with a large curvature or a steeper surface, contributing to a rough surface finish and compromised structural integrity (Fig. 4F) [29]. Comparatively, robotic arm-based could be a balanced moderation in dynamically adjusting contact force and angle, especially printing structures with complex surfaces.

### Overhang structure

3D printing fabricates parts layer by layer, so a previous layer must be built upon. In some instances, support structures should be created depending on how intricate the 3D model is. For instance, the upper features of the letter T need support from the model’s sides. Nothing can be used to print these out arms, and without supports, the material will collapse. However, by changing the part orientation, this structure can avoid any support materials, saving print time and improving print quality (Fig. 4G).

A threshold angle determines whether support is required on an overhang structure. To determine the region where the angle is bigger, a right triangle with the threshold angle is created between the preceding and current layers (Fig. 4H). The following equation, where *h* is the layer height and *θ* is the threshold angle, can be used to determine the length of the upper side of this triangle, denoted by the letter *d*.


$$\begin{array}{l} \begin{array}{l} d=h\cdot \tan\theta .\; \end{array} \end{array}$$

One of the dominant merits of robotic arm-based bioprinting lies in the capacity to print overhang structures by altering the operational printing path. Furthermore, it would be achievable to incorporate a reoriented platform or the extra axes, printing soft materials by aligning the extrusion layer with the underlying geometry to use it as support.

## Robotic arm-based advancements of *in situ* 3D bioprinted tissues and organs for reparative construction

Robotic arm based-3D bioprinting serves as an original approach in mediating defect repair in a more productive solution compared with conventional 3D bioprinting, where cell seeding and post-printing culture are highly required before transplantation. Notably, robotic arm-based *in situ* 3D bioprinting modalities are poised to directly deposit biomaterials precisely in the lesion site, which is time saving, especially in emergency circumstances. Moreover, a robotic arm-incorporated system endows flexibility, portability, and versatility according to distinct applicable situations. Therefore, various biomedical applications in terms of tissue repair have been explored to validate the dominant merits of robotic arm-based 3D bioprinting. This section will elucidate current advances in tissue engineering applications in cartilage, bone, skin, and muscle.

### Skin

**Figure 5.**
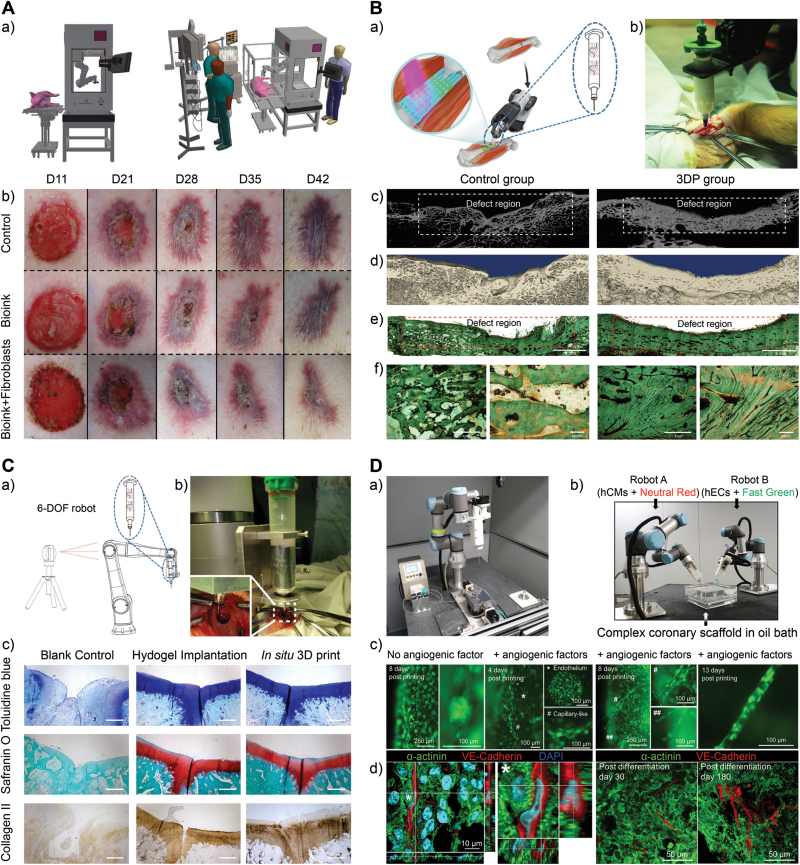
**Robotic arm-based 3D bioprinting strategies in tissue repair.**(A) Six-axis robotic arm-based intraoperative 3D bioprinting for severe burn injury repair. a) Configurative construction of a six-axis robotic arm-based bioprinter, BioAssemblyBot (BAB), and printing workflow. b) Characterization of skin closure under different experimental conditions (Scale bars: 1 cm). Reproduced with permission [22]. Copyright 2022, ASPS. (B) Robotic *in situ* 3D bioprinting for large segmental bone defect repair. a) Typical bioprinting and repair workflow. b) Bioprinting procedure during surgery. c) Micro CT scans of bone reconstruction at 12 weeks post-operation in control and 3DP groups. d) 3D reconstruction images of the control group and 3DP groups. e) Golden trichrome characterization. General views in control groups and 3DP groups. f) Magnified images of 40 × (left) and 100 × (right) in control groups and 3DP groups. Reproduced with permission [23]. Copyright 2021, Elsevier. (C) Application of robotic-assisted 3D bioprinter in cartilage application. a) Configuration of the 6-DOF robot. b) Printing process of *in situ* 3D bioprinting in the rabbit knee joint. c) The toluidine blue staining. d) Safranin O staining. e) Collagen II staining (scale bar: 500 μm). Reproduced with permission [30]. Copyright 2020, Elsevier. (D) Customized six-axis bioprinting system for vascularized cardiac tissue biofabrication. a) Overview of the 3D bioprinting platform. b) Setup of the two-robot collaborative bioprinting platform. Two 6-DOF robotic arm-based bioprinters are loaded with bioink containing either Neutral Red-stained human cardiomyocytes (hCMs) (colored in red) or Fast Green-stained human endothelial cells (hECs) (colored in green) to perform bioprinting procedures on a vascular scaffold. c) Artificial blood vessel cultured in the bioreactor for post-printing 8 days shows overgrowth of hCMEC/d3-eGFP cells (left) and its zoom-in view (right). Post-printed artificial blood vessels were cultured in the bioreactor with perfusion of angiogenic factors for 4 days, 8 days, and 13 days. d) Histological characterizations of the print-and-differentiated vascularized cardiac tissues. hCMs (α-actinin) and hECs (VE-cadherin) are detected in post-differentiation day 30 and day 180. Reproduced with permission [31]. Copyright 2022, Elsevier.

Skin, the largest organ in the human body, plays a pivotal role as a shielding barrier against possible mechanical and physical injuries or other external violations such as threatening substances. In the context of regeneration, the self-healing process of the skin is relatively restricted to the injury size, where a 4-cm wound in-diameter fails to regenerate without interventional strategies functionally. 3D bioprinting technique combined with robotic arms has proven a versatile toolbox in skin repair for dispensing specific cell types, bioink, and growth factors in a spatially controlled manner. Notably, robotic arm-assisted *in situ* bioprinting takes advantage of constructing irregular-sized wound areas with varying injury depth and topologies, which is conducive to being applied in clinical emergency settings. It is reported that a surgery-ready robotic-based 3D bioprinter could be utilized in a deep third-degree burn model via a six-axis robotic arm (Fig. 5A). This study validates the first proof-of-concept six-axis mediated 3D bioprinting strategy in skin repair, which exhibited positive performance in skin injury treatment [22].

### Bone

Bone defects can be brought on by a number of inherited or acquired disorders [32]. Globally, millions of bone transplant procedures are carried out annually to repair bone defects. Donor availability, graft incorporation, limited bioactivity, and high cost are the main drawbacks of conventional strategies for repairing lesion sites based on autogenous or allogeneic bone transplants. The most prevalent form of treatment for congenital problems is typically thought to be foreign-body implants, which functionalize in filling defect sites by enhancing mechanical strength and bioactive performances [33]. 3D bioprinting serves as a potential solution to tackling the concerns mentioned above. A viable pathway for employing 3D bioprinting in therapeutic settings is thought to be *in situ* healing of bone injury. Recent reports have demonstrated the feasibility of robotic arm-based *in situ* bioprinting strategies for tissue repair, which could also be a powerful intervention in bone regeneration.

The capability of a robotic platform (IMAGObot) to manufacture a 3D structure on various curved surfaces, such as bone models, was recently outlined. The robotic system was connected through a LinuxCNC-controlled FDM system. A path-planning algorithm that projects a 3D printing pattern onto the surface was created via MATLAB. It was revealed that the availability of 5-DOF makes it possible to dispense bioink on nonplanar surfaces [18]. Li *et al*. developed a robotic manipulator *in situ* 3D printer to repair long segmental bone defects in a swine model (Fig. 5B) [23]. The technical advancements realize functional restoration of long segmental defects within 12 min, where 3D bioprinted groups exhibited enhanced performance 3 months post-transplantation. More significantly, researchers have presented the progress in regenerative medicine via integrating 3D bioprinting and robotic-assisted minimally invasive surgery techniques. They validated the rationality of Remote Centre of Motion and extrusion-based bioprinting for restoring bone and cartilage defects at an average dimensional error of 0.06 ± 0.14 mm, which would be a promising tool for further application in the clinical setting [34].

### Cartilage

Osteoarthritis is one of the most common chronic joint diseases, resulting in inflammation and the degradation of articular cartilage [35, 36]. Cartilage regeneration is relatively tricky owing to the lack of vascularization and innervation in native cartilage. Applying a robotic-arm based 3D bioprinter in chondral defect repair is a promising strategy in terms of feasibility in repairing irregular defect areas *in situ* and with no requirements of pre-culture or incubation. For instance, Ma *et al*. have applied a 6-DOF-based robotic 3D bioprinter for *in situ* cartilage reconstruction and functional repair [30]. Further potential clinical applications are subsequently explored. In this study, the accuracy of the robotic arm could be significantly elevated, where the error of the printed surface is less than 30 μm, and the repair procedure was completed within 60 s. Chondrocytes in experimental groups are validated to be effective in biochemical and biomechanical properties (Fig. 5C).

### Other organs

Recent regeneration cases of other tissues and organs, such as cardiac and vascular tissues, have proven effective when incorporated with robotic arm-based 3D bioprinting techniques. For instance, Zhang et al. developed a six-axis robotic arm-based 3D bioprinter to realize vasculogenesis and angiogenesis of bioprinted blood vessels as well as the long-term survival of bioprinted cardiac tissues via single- or multi-robot operation (Fig. 5D) [31]. In this work, researchers designed a six-DOF robot arm to support multidirectional cell printing compared with the cartesian bioprinter. Apart from single robot arm operation, a multi-arm collaborative platform is also demonstrated to be practical and advantageous at printing onto complex-shaped scaffolds. From the perspective of six-axis 3D bioprinting, the six-DOF robot is equipped with outstanding spatial solutions to reach any coordinate in theory, thus, printing cells in any position of complex shape is possible [37, 38]. Recent advances in robotic arm-based 3D bioprinting for tissue engineering and regenerative medicine have been summarized in Table 3.

**Table 3.** Summary of the latest research on robotic arm-based 3D bioprinting for tissue engineering and regenerative medicine.

Printer type	Controlled attachment	Outcomes	Application	Ref.
Six-axis robotic arm	Closed-loop tool centre point (TCP) calibration method	Cartilage injury healing 12 weeks post-printing	Cartilage defect repair	[30]
Ferromagnetic soft catheter robot	Magnetic actuation	1. High printing accuracy and fidelity 2. Minimally invasive for internal organs	1. Porcine tissue surface bioprinting 2. Rat liver surface bioprinting	[39]
Extrusion-based robotic arm	Open loop computerized tomography (CT) scan	1. Improvement in bone structure and mechanical strength 2. Regenerative bone tissue in the defect site	Large segmental bone defect in the porcine model	[23]
Multi-arm dispensing system/micro-solenoid valve system	Open-loop selective laser sintering (SLS) scanning/laser scanner	1. Bone tissue formation 2. Faster wound healing efficiency 3. Accelerated closure of dermis for soft tissue	Hard and soft tissue reconstruction	[40]
Six-DOF robot bioprinter	In-house developed C++ scripts	1. Vasculogenesis and angiogenesis of bioprinted blood vessels 2. Long-term survival of bioprinted cardiac tissues	1. Cardiac tissue construction 2. *In vivo* organ developmental process mimicking 3. *In vitro* biofabrication of complex organs	[31]
Deployable extrusion-based bioprinter “BioArm”	Home-written Python codes	1. *De novo* synthesis of extracellular matrices 2. Enhanced cellular proliferation compared to the tumor alone 3D printed spheroid culture	1. Tumor microenvironment research 2. Drug testing for cancer	[41]

## Challenges of robot arm-based bioprinting in clinical applications: from bench to bedside

**Figure 6.**
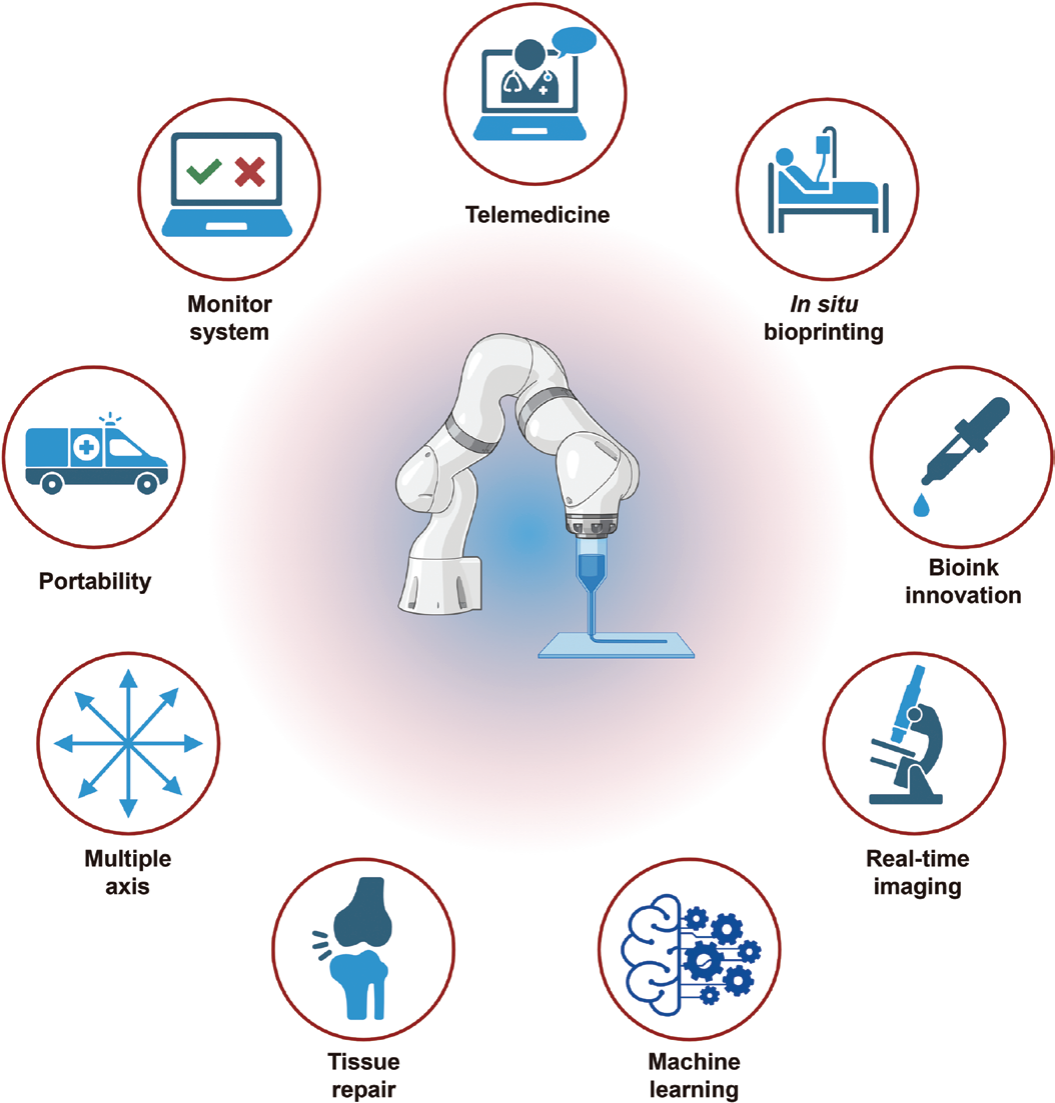
Graphical diagram of future orientation of robotic-arm-based 3D bioprinting techniques in regenerative medicine.

Compared with conventional bioprinting modalities, the integration of robotic arms implies a dimensional escalation of the degree of freedom, which is a blessing in enhancing bioprinting complexity and flexibility but also issues of sophistication in the scope of mechanical engineering. Specifically, a robotic arm-based 3D bioprinter allows for multiple printing patterns on curved or dynamic interfaces; a synergistic collaboration of robot arms would be an ideal solution in biofabricating organs or tissues with intrinsic gradients. On the other hand, robotic arm-based surgical facilities tend to maturate via consistent applications in medical cases. For instance, SpineAssist®, the first and the most broadly utilized spinal surgery robot, plays a pivotal role in pre-specified trajectory design to enhance surgical precision, which the Food and Drug Administration approved in 2004 [41]. Besides, Mazor X® stealth robotics are developed to facilitate predictability, controllability, and flexibility in operative cases [42]. Nevertheless, given robotic arm-based 3D bioprinting, this technique is in its infancy for future treatment regimens and clinical applications, followed by thorough pre-clinical evaluation. Notably, researchers have developed *in situ* bioprinting robots coupled with endoscopes to treat gastric wall defects at the microscale, which shed light on prospects for clinical applications. However, quite a few concerns should be addressed for future clinical translation, which will be discussed in this section as follows (Fig. 6).

### Fundamental design and development

The evolution from conventional cartesian 3D bioprinters to articulated robotic arm 3D bioprinters will inevitably raise the complexity of the system, cascading various challenges in aspects of design and development.

Firstly, current commercial 3D bioprinters use the same setup as common cartesian 3D printers in general, with modifications in different content, such as using biomaterials as ink and temperature control to keep bioactivity or maintain the properties of biomaterials during the printing process. In other words, the current development of 3D bioprinters is alterations of specific applications of biological and medical scenarios based on the mature market of 3D printers. The rapid iterations of this market contributes to a positive operating system development, thus implying the user interface (UI) is friendly to new beginners. Secondly, the slicer software is integrated into the bioprinter, and the slicer patterns are well adapted for the cartesian layer-by-layer 3D printing method. Moreover, printing path designs are auto-generated with a graphical user interface without the programming expertise of the primary users of 3D bioprinters, researchers, and doctors.

Specifically, there are several robot arms manufacturers, each of which has its unique robot controller with diversified controller software. Printing job software for 3D bioprinters must be developed according to a myriad of controller software, which hinders industrial standardization and research universality for regular users. The intricacy of the software also increases R&D time and expense. Meanwhile, slicing 3D model with slicer software is a priority for the accomplishment of the printing job, while current robotic arm-based bioprinting rigidly adapts their software from previously established cartesian 3D printing system or merely develop their slicer strategy with no flexible customization. In this case, distinct printing jobs could not fit smoothly in robotic arm-based 3D bioprinting. Not to mention, the current programming language for printing path planning, G code, is tricky to be adaptable.

On the other hand, the precision and quality of 3D printing are strongly impacted by the rigidity of the printer structure. To construct high-quality prints, a 3D printer must move the print nozzle rapidly while retaining sub-millimeter positional accuracy. Creating a structure surrounding the print area is considerably simpler than creating one that stands off to the side and has an arm reaching over to perform the printing. Precisely, the robot arm’s damping control cannot be sufficiently controlled by the motor control alone. The deviation scaled up by the complexity of the multi-axis system and the vibrations generated by the machine itself will produce a notable impact on the resolution of printing. The issue of positioning accuracy will be escalated while doing delicate 3D bioprinting tasks.

Furthermore, the cooperation between multiple robot arms, such as 3D bioprinting a heart with multiple bioinks, comes with a complicated directional printing procedure program, which will significantly increase the difficulty in designing and developing the algorithm of robot kinematics, avoiding collision, and adaptive operation procedure development according to various situation changes.

### Multi-technique collaboration

From the developmental paces of robotic arm-based technology, even though robotic-assisted systems are conducive to operative accuracy, satisfactory automation, and compatibility with minimally invasive incisions, further applications in clinical contexts remain to be tackled. Primarily, the robotic arm-based bioprinting technique is prototypically in its infancy, where dimensional techniques shall be synergistically collaborated and optimized, such as defect scanning, programmable interface, and printing trajectory customization. In addition, human-controlled robotic interventions during intraoperative tasks require specialized expertise. Also, *in situ* bioprinting techniques are presently confined to areas close to the skin, which requires the appropriate intervention of surgeries for printing on internal organs or tissues.

### Bioink selection and cell sources

Controllable cell suspension prior to direct *in situ* bioprinting is required in tissue biofabrication. In contrast, ethical approval toward stem cell expansion and subsequent human employment remains to-be-solved [43]. In many countries, owing to the uncontrollable differentiation potential and possible tumorigenic risk of embryonic stem cells, further utilization in clinical applications for *in situ* 3D bioprinting can be hindered [44]. Moreover, stem cell culture protocols and procedures may suffer from variability, giving rise to undesirable cell differentiation. More importantly, transplanted cells or biochemicals for biological induction might trigger an immune response or rejection in the host-tissue interface, which hamper further clinical translation of *in situ* 3D bioprinting. It is worth addressing that pre-vascularization may be required in specific tissue or organ regeneration cases, such as the heart and liver. The existing challenge resides in the pre-culture or cell pre-loading procedures, which may be time-consuming, especially in an emergency.

Given the optimization of bioink, current bottlenecks could lie in potential harm for human organisms brought by specific crosslinking strategies, such as UV curing, thermal crosslinking, or chemical emersion, in which case, residing healthy tissues may be influenced to some extent, especially when printing on deep inner organs. Moreover, how to moderate relative rates of ECM deposition and biomaterial degradation should be taken into consideration in preliminary bioink design.

### Ethical concerns and regulatory issues

Despite the remarkable strides of 3D bioprinting in tissue construction, upcoming ethical concerns and regulatory issues should also be addressed. For instance, conducting *in situ* bioprinting on the human body might raise biosafety concerns [45]. Various rules and regulations should also be enhanced for tissue repair and regeneration in clinical applications [46]. Apart from that, the financial scope, affordability, law enhancement, and decent insurance policies might boost further popularization and availability of this technology.

## Future perspectives and outlooks

Although robotic arm-assisted bioprinting techniques have been applied in tissue engineering and regenerative medicine, the potential of robot arms is not yet fully developed and integrated. Based on automated arm-based surgery robots in clinical surgeries and *in situ* bioprinting applications, advanced functions such as obstacle avoidance, artificial intelligence (AI)-integrated algorithms, real-time monitoring, and feedback systems shall be synergistically developed to enhance the versatility of robotic arm-based bioprinting techniques in biomedical applications. In this section, specific future orientations will be relatively discussed as follows, and future perspectives of robotic arm-based 3D bioprinting are illustrated in Fig. 5.

### Digital revolution

With technological revolutions such as 5G and AI, healthcare and biomedical industries could be profoundly revolutionized. AI-based algorithms and big data could be practical solutions to the abovementioned concerns. A few attempts have been undertaken to develop an AI-powered application for generating a simulated 3D printable model from CT scan data, which would enable the printing process to be adequately designed. Furthermore, incorporating AI-based techniques endows labor-free clinical intervention, in which case, time-consuming issues could be ameliorated. On the other hand, AI coupled with 3D bioprinting is projected to optimize performance at all phases of the bioprinting workflow, encompassing preparation, bioprinting, and post-bioprinting, with fewer human errors and a less susceptible to errors overall bioprinting workflow. In reality, an alternative but efficient approach is prone to be the one that promptly converts current magnetic resonance imaging (MRI)/confidence interval-based 3D models into cell-based models. In this case, AI algorithms intelligently allocate cells and tissue properties to a virtual organ model with spatial precision and material multiplicity. The slicing process for layer-by-layer bioprinting fashion should also be integral cell-based matching extrusion parameters, which quite differs from conventional slicing for 3D bioprinting. Apart from generating the printing path, AI exhibits great potential in image recognition for smart calibration before the printing procedure and especially for the intervention of robotic arms. AI-empowered adaptive force feedback would automatically adjust the printing interfaces between printing tips and curved surfaces with complex topological properties. In this scenario, AI would be a powerful helper to surgeons and scientists. Besides, 5G could be a practical helper in handling teleoperation to accomplish scalability and accessibility of robotic arm-assisted 3D bioprinting in most areas.

Moreover, non-invasive surgeries could benefit from developing advanced mini-robots, which can be integrated with 4D bioprinting techniques to alter morphologies or functionalities over time. Biodegradable robots loaded with bioink would be advantageous for repairing particular internal tissues or organs. Interdisciplinary interactions between diverse research fields are imperative to stimulate additional breakthroughs and advance therapeutic applications.

The breadth of *in situ* bioprinting has been constrained by dynamically altering geometries, i.e. moving surfaces due to respiratory or twitchy motions. Zhu *et al.* have formulated an adaptive extrusion-based printing technology with closed-loop feedback and a computer-vision-based control system allowing for real-time rectification of printing errors produced by the fluctuant printing surface [15].

### Technical integration

The acquirement of high-definition and precise scanning data in the lesion site is the prerequisite before conducting *in situ* robotic arm-based 3D bioprinting. Commercial 3D scanners such as ZScanner™ Z800 scanner (0.05 mm) and EinScan-Pro 2 × (0.2 mm) are available compared with CT and MRI for excellent spatial resolution [47]. Real-time data can be supplied by integrating with fast 3D scanners, which could decrease printing errors and enable instant corrections. Additionally, instantaneous quality control can be achieved while manufacturing a tissue construct by integrating online monitoring and inspection sensor systems. In this context, the built-in machine visual system can enable monitoring of the constructed geometry and the printing fidelity. This approach may also offer the intriguing possibility of rapidly eliminating the noncompliant tissues to fix the bioprinting procedure. To examine and control the bioprinting setting, inspection sensors such as temperature, CO_2_ and O_2_, pH, and humidity sensors are crucial [48].

### Axis escalation: from six-axis to seven-axis

In principle, 3D bioprinters with multiple axes could be regarded as the next significant milestone of the 3D bioprinting industry for the following reasons. First, multiple axes allow for a more complicated design. The printer can accurately capture irregular geometries due to its improved mobility, which presents significant alternatives for printing on curved surfaces. Second, more axes mean fewer materials and faster speed. There is little to no requirement for support structures given the axial motion of the printers, which varies on the specific printer. Fewer support results in speedier printing and, of course, uses less material. Third, as for printing path planning, such a robotic device may contain software that could automatically interpret the geometry of the object and generate a tool path that was significantly more efficient.

However, there are inevitable issues regarding more axes in robotic arm-assisted 3D bioprinting. Conventional 3D bioprinters can achieve satisfying printing resolutions in less than 100 microns. For robot arms, the accurate positioning of the tool head is insufficient, especially in the cheaper versions. That is, robot arms are more qualified to handle large-sized tasks compared with minor scales. Besides, even though robot arms have been widely applied in the industry, it is rendered to be more expensive than conventional 3D bioprinters.

## Concluding remarks

The capability of robotic arm-based 3D bioprinting techniques to regenerate cartilage, bone, skin, cardiac, and vascular tissues in distinct injury models has been acknowledged. This review examines the latest developments in robotic-assisted 3D bioprinting technology in 3D bioprinting, where the fundamental modalities of robotic arms are explored, as well as robot configurations and applications in tissue engineering. With the rapid improvement of knowledge and technology in interdisciplinary fields, robotic arm-assisted 3D bioprinting is expected to be practical in the future.
